# Rapid degradation of malachite green by CoFe_2_O_4_–SiC foam under microwave radiation

**DOI:** 10.1098/rsos.180085

**Published:** 2018-06-27

**Authors:** Yanpeng Mao, Shanxiu Yang, Chao Xue, Miaomiao Zhang, Wenlong Wang, Zhanlong Song, Xiqiang Zhao, Jing Sun

**Affiliations:** National Engineering Laboratory of Coal-fired Pollutants Emission Reduction, School of Energy and Power Engineering, Shandong University, Jinan 250100, People's Republic of China

**Keywords:** microwave, CoFe_2_O_4_–SiC foam, organics, degradation, continuous treatment

## Abstract

This study demonstrated rapid degradation of malachite green (MG) by a microwave (MW)-induced enhanced catalytic process with CoFe_2_O_4_–SiC foam. The catalyst was synthesized from CoFe_2_O_4_ particles and SiC foam by the hydrothermal method. X-ray diffraction and scanning electron microscopy techniques were used to confirm that CoFe_2_O_4_ particles were settled on the surface of SiC foam. In this experiment, a novel fixed-bed reactor was set up with this catalyst for a continuous flow process in a MW oven. The different parameters that affect the MW-induced degradation rate of MG were explored. The MW irradiation leads to the effective catalytic degradation of MG, achieving 95.01% degradation within 5 min at pH 8.5. At the same time, the good stability and applicability of CoFe_2_O_4_–SiC foam for the degradation process were also discussed, as well as the underlying mechanism. In brief, these findings make the CoFe_2_O_4_–SiC foam an excellent catalyst that could be used in practical rapid degradation of MG.

## 1. Introduction

Rapid industrial and economic development could increase the risk of environmental pollution. In particular, industries such as paper mills, pharmaceutical production, textile printing and dyeing industry can produce persistent pollutants in waste water that are difficult to degrade after discharge. These persistent pollutants are very harmful to wildlife and humans. As a representative organic dye, malachite green (MG) is usually used in the tinting of silk, leather and paper, and its triphenylmethane structure is toxic. Therefore, MG poses a major risk in the soil environment, with the possibility of eventual ecosystem disruption [1]. Studies have shown that MG cannot undergo efficient biodegradation [2]: it can stay in the muscle and organs of catfish for as long as ten days. Moreover, it will contaminate most animals that are in contact with it [3–5].

Because of the potential ecological damage from organic pollutants, many researchers have investigated their removal methods.

In the traditional treatment of organic solutions, the adsorption method is commonly used. The characteristic of the adsorption method is to transfer the organic matter in the water from the water phase to the solid phase, but the structure of the organic matter itself does not change. Sharma *et al*. [6] synthesized starch/poly (alginic acid-cl-acrylamide) nanohydrogel (ST/PL(AA*-*cl*-*AAm)NHG) by the polymerization method. The study found that this nanohydrogel was effective for removal of coomassie brilliant (CB) blue dye from aqueous solution and the adsorption of CB onto ST/PL(AA*-*cl*-*AAm)NHG was immensely dependent upon various parameters. Javadian *et al*. [7] synthesized polyaniline/g-alumina nanocomposite by the chemical oxidation method. Batch adsorption experiments were conducted for removing three types of hazardous dyes, Reactive Red 194, Acid Blue 62 and Direct Blue 199, from aqueous solution and the effects of pH, adsorbent dosage, contact time and initial concentration of dyes were investigated. The results showed that the polyaniline/g-Al_2_O_3_ nanocomposite could be used as a suitable adsorbent for textile waste water purification. In addition, compared to the traditional methods, in recent years, the microwave (MW) irradiation method as a new way to eliminate organics in water has become a hot topic. MW irradiation, an electromagnetic wave with a frequency range from 300 MHz to 300 GHz [8], has been used for heating since 1937. Compared with conventional heating methods [9], MW irradiation has a higher heating rate and exhibits selective and volumetric heating properties [10]. Owing to these features, it has been widely studied for removing organics from wastewater, especially during recent years. The topics studied include the catalyst, the equipment used for degradation and different ways to apply MW irradiation.

There are different methods of applying MW irradiation to treat waste water. They can be categorized into Fenton/Fenton-like process, catalytic wet air oxidation and combinations of MW radiation with other physical fields. Particularly, MW-induced catalytic degradation (MICD) and MW-induced catalytic oxidation (MICO) are the most popular processes. In MICD, MW irradiation is combined with a suitable MW absorbent (catalyst), and in the MICO process MW irradiation is combined with a suitable MW absorbent (catalyst) and oxidants.

As the absorption of MW radiation is selective [11], the appropriate materials should be used to degrade organics. The catalyst as a key factor has also been studied widely. In recent years, MW absorbents such as metallic particles [12], active carbon (AC) [13] and polymers [14] have been examined for waste water treatment. Ghaedi *et al*. [15] used AC loaded with zinc oxide nanoparticles to degrade MG. In addition to the effects of initial dye concentration, pH and absorbent dosage, the authors also studied the kinetics and found that the adsorption of MG follows a pseudo-second-order rate equation. An MG degradation rate of more than 95% was achieved. Gao *et al*. [16] loaded MgFe_2_O_4_ on SiC to degrade the azo dye Direct Black BN. The high degradation efficiency (96.5% after 5 min) was attributed to the MW-induced ·OH radicals and holes. Chen *et al*. [17] used carbon nanotubes as the catalyst in the degradation of methyl parathion and methyl orange. Complete degradation was achieved within 7 min with an irradiation power of 450 W at pH = 6.0. Although these studies showed very high degradation rates, several problems remained. The catalyst particles used were fine and therefore difficult to separate from waste water. Moreover, most of these processes are not continuous, which lowers the reaction efficiency. Bo *et al*. [18] used carbon-supported copper as a catalyst in a continuous reaction process: waste water flowed through a catalyst-filled reactor, and after 5 h of operation the degradation rate of *p*-nitrophenol (PNP) was 91.8%. Longli Bo *et al*. [19] investigated the degradation of PNP using granular activated carbon as a catalyst; the degradation rate reached 90% after 2 h of MW irradiation. Zhang [20] synthesized coal fly ash/CoFe_2_O_4_ (CFA/CFO) composites for the removal of MG from aqueous solution. The adsorption of MG onto CFA/CFO fitted quite well to the Freundlich and DKR isotherm models. The removal efficiency was 90% by MW irradiation, indicating that the composite could potentially be used for the efficient removal of MG from water.

It is well known that SiC foam has good adsorbability and porosity, as well as excellent MW absorption. In this experiment, we used SiC foam as a skeleton and loaded it with CoFe_2_O_4_ particles, which are also good absorbers of MW radiation. Using MG as a representative organic pollutant in waste water, the CoFe_2_O_4_–SiC foam was placed in a fixed bed as the catalyst to degrade MG under MW irradiation. Gao *et al*. [16] used granular SiC loaded with MgFe_2_O_4_ on the surface; the SiC foam with CoFe_2_O_4_ had good porosity and is suited for a continuous reactor. In this article, the degradation efficiency and activity of the prepared catalysts were investigated. The stability and applicability of CoFe_2_O_4_–SiC for use in the degradation process were also discussed, and a possible degradation mechanism was proposed.

## 2. Material and methods

### 2.1. Preparation of catalysts

SiC foam was purchased from Jingxian County Hengtong casting material factory; this is used to filter liquid iron in industry. Iron(III) nitrate nonahydrate (Fe(NO_3_)_3_·9H_2_O), cobalt nitrate hexahydrate (Co(NO_3_)_2_·6H_2_O), absolute ethanol and hydrofluoric acid were purchased from Sinopharm Chemical Reagent Co., Ltd MG was obtained from Zhiyuan Chem. Co., Ltd (Tianjin, China). Sodium hydroxide (NaOH) was purchased from Yili Fine Chem. Co., Ltd (Beijing, China). Apart from the SiC foam, all the chemicals were of analytical grade and used without further purification. Ultrapure water was used throughout the study.

### 2.2. Catalyst and characteristics

The CoFe_2_O_4_–SiC foam catalyst was synthesized by a hydrothermal method. The SiC foam was washed with distilled water several times and dried at 80°C in air for 4 h. Fe(NO_3_)_3_·9H_2_O (0.005 mol) and Co(NO_3_)_2_·6H_2_O (0.0025 mol) were dissolved in 200 ml of distilled water. After 10 min of stirring, 10 ml of NaOH solution (3.5 mol l^−1^) was added, and the mixed solution was stirred for 30 min afterwards. The suspension and the SiC foam were transferred to a 250 ml Teflon-lined autoclave, and heated at 150°C for 12 h. After cooling to room temperature, the foam was taken out and washed with distilled water and absolute ethanol several times, and then dried at 80°C in air for 4 h. Finally, the foam was placed in a muffle furnace and heated at 550°C for 8 h. For comparison, we also prepared CoFe_2_O_4_, using the same method with 6.25 × 10^−4^ mol Fe(NO_3_)_3_·9H_2_O and 3.125 × 10^−4^ mol Co(NO_3_)_2_·6H_2_O. The amount of CoFe_2_O_4_ loaded on the foam was determined by the weight change in SiC.

### 2.3. Experimental methods

**Figure 1.**
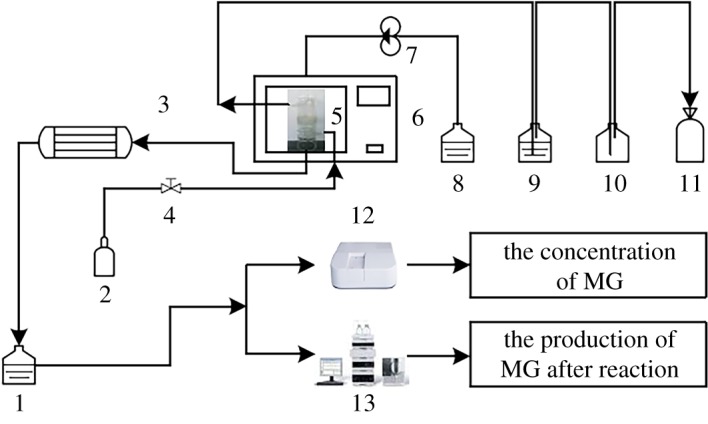
Diagrammatic sketch of experimental set-up equipped with the microwave system (1. Solution container, 2. Gas cylinder, 3. Condenser tube, 4. Valves, 5. Fixed bed quartz reactor, 6. Microwave oven, 7. Peristaltic pump, 8. Solution container, 9. Gas washing bottle, 10. Gas washing bottle, 11. Gas collection bottle, 12. Ultraviolet spectrophotometer and 13. LC-MS).

MG was selected as the target pollutant rather than real industrial waste water to investigate the performance of the synthesized CoFe_2_O_4_–SiC catalysts. The experiment was carried out in a simple fixed-bed reactor; the experiment flow chart is as shown in figure 1. Firstly, four pieces of the CoFe_2_O_4_–SiC catalyst were put into the reactor and the system was washed using distilled water for 5 min. Secondly, the microwave oven (M3-L233C, 2450 MHz, Midea, China) and gas cylinder were opened simultaneously at 900 W for 60 s to dry the system. Afterwards, MG solution was pumped into the reactor by a peristaltic pump which could control the flow speed; meanwhile a microwave oven and cooling system were switched on. In the middle of the reactor containing the CoFe_2_O_4_–SiC catalyst, the pollutant in the waste water was degraded by a combination of catalyst and MW irradiation. The exhaust gas was first passed through the gas absorbing bottles and collected using a gas collection bottle. At the other end of the reactor, a condenser tube was connected with the reactor to cool the solution heated by the MW irradiation. The solution after cooling was collected, and the residual MG and the degradation products were measured using the spectrophotometry method and high-performance liquid chromatography, respectively.

The degradation rate (*R*) by the MW catalytic reaction was calculated by the following equation:

2.1 $$R=\frac{\left(C_{0}-C\right)}{C_{0}}\times 100\%,$$

where *C*_0_ is the original concentration of MG and *C* is the concentration of MG after the reaction.

The experiment parameters were varied in the range of 2.5–10.5 for pH, 10–25 ml min^−1^ for flow rate, 90–900 W for MW power and 20–50 mg l^−1^ for MG initial concentration.

### 2.4. Instrumentation

The morphology and surface characteristics of SiC and CoFe_2_O_4_–SiC were observed with a Shimadzu EPMA-1600 Electron Probe Microanalyzer. The powder X-ray diffraction (XRD) spectra of the catalyst were obtained (LabX XRD-6000, Shimadzu, Japan) with Cu KR radiation. The pH values of the MG solutions were adjusted using a PHS-3C pH meter (Shanghai, China) combined with a glass electrode. The concentration of MG was measured by an ultraviolet spectrophotometer (TU-1901, Persee, Beijing, China). The degradation product from the reactor was measured by liquid chromatography–mass spectrometry (LC-MS; Agilent-6100, Germany).

## 3. Results and discussion

### 3.1. Characterization of CoFe_2_O_4_–SiC

**Figure 2.**
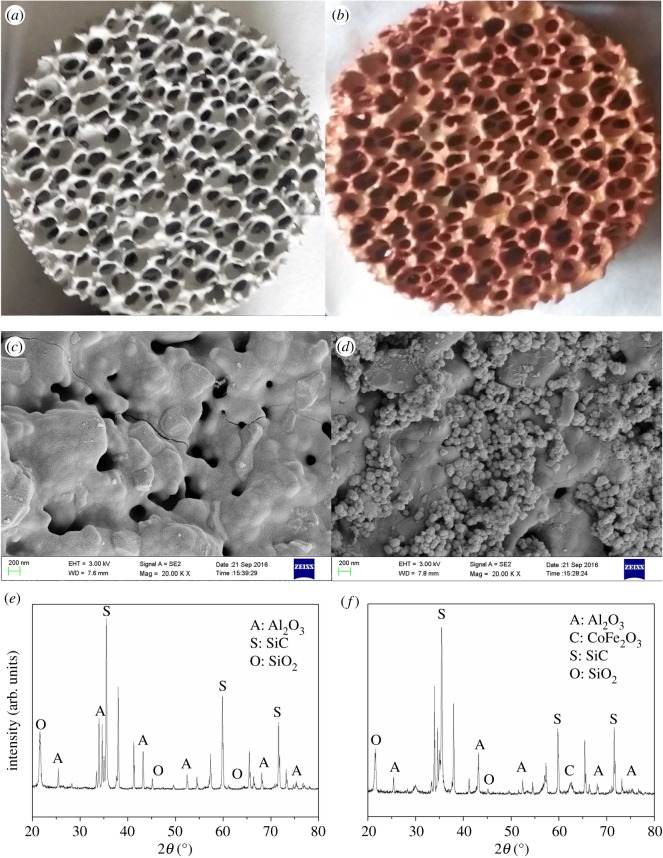
Photographs of (*a*) SiC and (*b*) CoFe_2_O_4_–SiC foams. SEM images of (*c*) SiC and (*d*) CoFe_2_O_4_–SiC foams. XRD patterns of (*e*) SiC and (*f*) CoFe_2_O_4_–SiC foam.

XRD patterns, scanning electron microscopy (SEM) images and X-ray photoelectron spectral features were used to study the crystal structure and morphological feature of the CoFe_2_O_4_–SiC foam. Figure 2*a,b* shows photographic images of the SiC and CoFe_2_O_4_–SiC foams, respectively. As shown in figure 2*c*, before loading the catalyst, the SEM image indicates that the surface of SiC was porous and smooth, and the porous structure could facilitate the adsorption of CoFe_2_O_4_. Figure 2*d* shows the SEM image after loading the CoFe_2_O_4_ particles (shown as spheres attached to the apertures). These results suggest the successful deposition of catalyst microparticles. As shown in figure 2*f*, the prepared CoFe_2_O_4_–SiC has sharp XRD peaks, showing a CoFe_2_O_4_, crystal pattern which is in agreement with previous reports [21].

**Figure 3.**
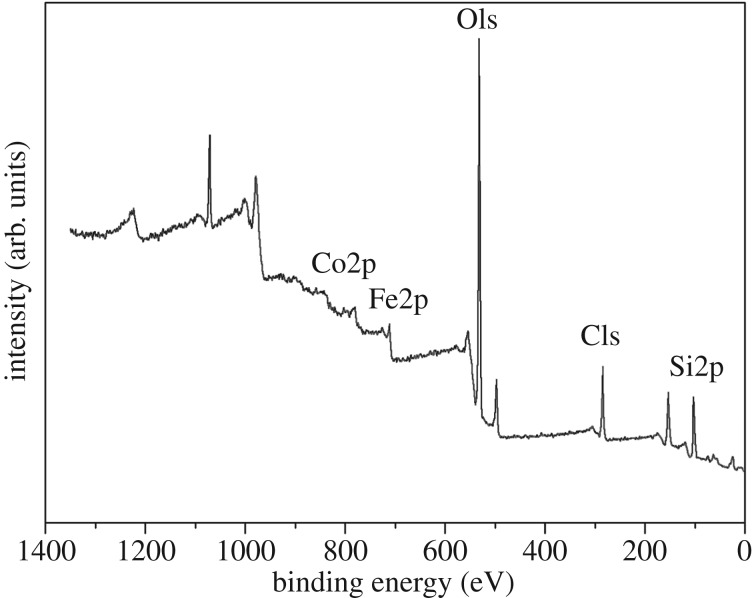
X-ray photoelectron survey spectrum of CoFe_2_O_4_–SiC foams.

The chemical composition and electronic structure of the synthesized CoFe_2_O_4_–SiC foam are given. As observed in figure 3, the survey spectrum of CoFe_2_O_4_–SiC foam displayed peaks of Co, Fe, O, C and Si. The peaks at 539.7 eV (O 1 s), 110.2 eV (Si 2p), 292.2 eV (C 1p), 735.7 eV (Fe 2p) and 810.12 eV (Co 2p) are shown in electronic supplementary material, figure SF-1. It confirmed the presence of CoFe_2_O_4_, loaded on the surface of the SiC foam.

### 3.2. Activity of CoFe_2_O_4_ in microwave-assisted catalytic reaction

**Figure 4.**
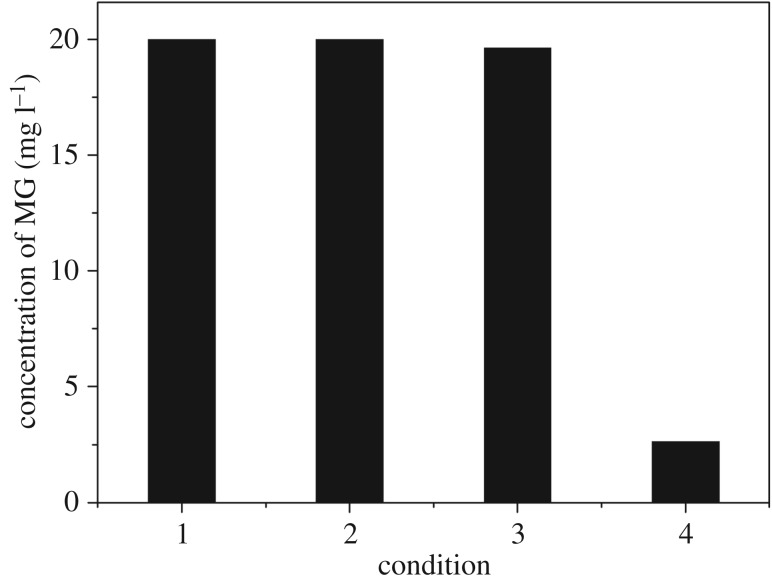
Concentration of MG under different conditions: (1) with neither irradiation nor catalyst, (2) without catalyst, (3) without irradiation, and (4) with both irradiation and catalyst. Original MG concentration: 20 mg l^−1^, current speed: 10 ml min^−1^, pH: 4.5, and MW irradiation: 900 W.

The activity of CoFe_2_O_4_ was examined both in the absence and presence of MW irradiation. As shown in figure 4, with MW and without the catalyst, the solution passing through the reactor maintained the initial MG concentration (20 mg l^−1^). The same was found with the catalyst and without MW (final MG concentration: 19.625 mg l^−1^); it can be seen that SiC foam had no effect on the adsorption of MG solution. However, when both the MW radiation (900 W) and the catalyst were present, the MG concentration was dramatically reduced to 2.627 mg l^−1^. Therefore, both MW irradiation and CoFe_2_O_4_–SiC foam are required to effectively remove MG from the water.

### 3.3. Catalyst performance under different operation conditions

#### 3.3.1. Influence of initial pH

**Figure 5.**
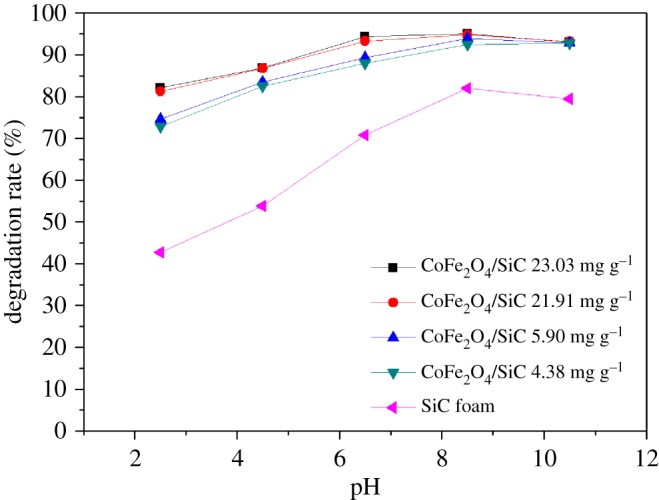
Effect of initial pH with different CoFe_2_O_4_ loadings. Initial concentration of MG: 20 mg l^−1^, MW irradiation power: 900 W and current speed: 10 ml min^−1^.

The effect of initial pH on the degradation rate of MG is shown in figure 5. The pH of the original MG solution was 4.5, and it was then adjusted to various pH values between 2.5 and 10.5 by using HCl and NaOH. In general, MG removal rate was enhanced as the pH increased from 2.5 to 8.5, and then declined slightly between pH 8.5 and 10.5. In the range of 2.5–8.5, the degradation rate of MG increased from 82.1% (pH = 2.5) to 95.01% (pH = 8.5), and the degradation rate of MG declined from 95.01% (pH = 8.5) to 93.07% (pH = 10.5) when the loaded ratio of CoFe_2_O_4_/SiC was 23.03 mg g^−1^. Increasing the pH was evidently an unfavourable factor for the removal of MG. This is attributed to the influence of pH on the surface charge of the catalyst in the solution; the prediction of actual charge on CoFe_2_O_4_ is challenging due to the different pH of the MG solution. On the other hand, around pH 8.5, more of ^.^OH species was generated according to equations (3.6)–(3.9), which would increase the degradation rate and efficiency of MG. With regard to the SiC foam, because of the SiC foam contained a small amount of metal elements, such as aluminium and iron, there was still the Fenton-like reaction on its surface. Therefore with the increase of pH, the reaction was promoted and the degradation rate of MG reached its highest at pH = 8.5.

#### 3.3.2. Influence of microwave power

**Figure 6.**
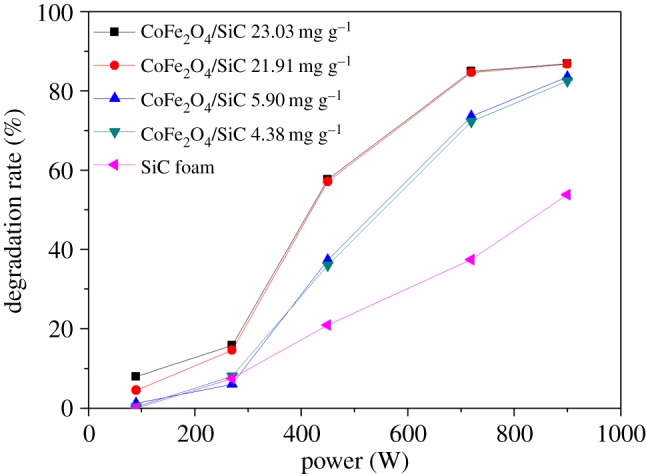
Effect of MW power with different CoFe_2_O_4_ loadings. Initial concentration of MG: 20 mg l^−1^, initial pH: 4.5 and current speed: 10 ml min^−1^.

The power of MW irradiation is an important operating parameter in MW-assisted catalytic reactions. In this experiment, five different power settings of the microwave oven were used (90, 270, 500, 720 and 900 W), while other conditions were fixed (pH: 4.5, flow rate: 10 ml min^−1^ and initial MG concentration: 20 mg l^−1^). As shown in figure 6, the degradation rate of MG by CoFe_2_O_4_–SiC increases monotonically with the MW power in the range of 90–900 W. There are also two turning points at 270 and 720 W, between which the MG degradation changes the fastest with MW power (i.e. steepest slope). When only SiC foam was used, on the other hand, the MG degradation ratio changes at the same rate with increasing MW power. There are two potential mechanisms for the enhanced MG degradation. First of all, a higher MW power leads to a higher temperature, which inevitably accelerates the degradation reaction. Secondly, more hotspots are generated as more MW energy is absorbed by CoFe_2_O_4_, thereby producing more catalytically active sites on the CoFe_2_O_4_ particle surface. At the same time, the SiC foam can effectively absorb the MW irradiation, and the increased degradation of MG on the surface of SiC foam alone is mainly attributed to the temperature change. Therefore, in subsequent experiments we used the MW power of 900 W and the CoFe_2_O_4_–SiC catalyst, unless noted otherwise.

#### 3.3.3. Influence of initial malachite green concentration

**Figure 7.**
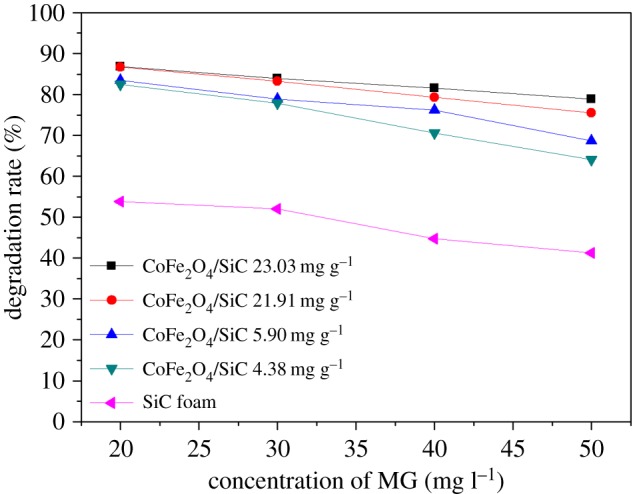
Effect of initial concentration of MG with different CoFe_2_O_4_ loadings. Initial pH: 4.5, current speed: 10 ml min^−1^, MW irradiation power: 900 W.

The effect of initial MG concentration in the range of 20–50 mg l^−1^ on the degradation rate was investigated. As shown in figure 7, the degradation rate of MG decreased with its initial concentration. In this experiment, the MG solution was moving through the reactor while being irradiated by MW. The MG then made contact with the catalyst on the surface of the CoFe_2_O_4_–SiC foam. When the MG concentration was high, the number of hotspots on the surface was insufficient for degrading the MG in time, hence the reduced degradation rate. Therefore, the concentration of MG in the solution is another important parameter of the reaction.

#### 3.3.4. Influence of flow speed

**Figure 8.**
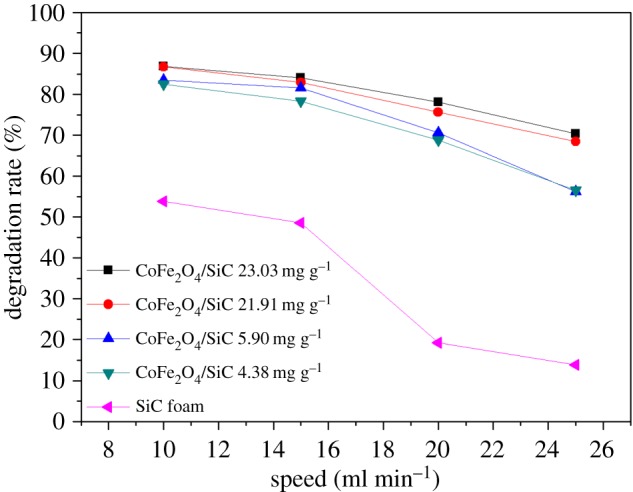
Effect of current speed with different CoFe_2_O_4_ loadings. Initial concentration of MG: 20 mg l^−1^, initial pH: 4.5 and MW power: 900 W.

Unlike previous studies, the reactor here was designed to allow the MG solution to flow through at a rate (10–25 ml min^−1^) controlled by a peristaltic pump. As shown in figure 8, the degradation rate of MG decreased with rising current speed. Particularly, when only SiC foam was used, the degradation rate dropped sharply at 15 ml min^−1^. As the MW radiation heats water in the reactor, a higher flow rate means less temperature rise in the reactor, which could have a huge impact on the reaction. The CoFe_2_O_4_ particles loaded on the SiC foam could absorb more MW radiation and raise the temperature even further. This could offset the reduced temperature rise and prevent the MG degradation rate from dropping rapidly. The effect of current speed indicates that, in a continuous-mode reaction, a slower speed of solution flow is beneficial for the reaction.

#### 3.3.5. Influence of CoFe_2_O_4_ loading

The degradation rate was enhanced with more CoFe_2_O_4_ catalyst, as it provides more active sites on the surface of SiC foam in contact with MG during the MW-assisted catalytic reaction (figures 5–8). However, as the amount of CoFe_2_O_4_ particles increased beyond a certain threshold, the particles start to overlap, causing no obvious increase of effective contact with MG. Considering that the power of MW irradiation and the current speed both affect the reaction, the amount of CoFe_2_O_4_ should be adjusted accordingly.

### 3.4. Catalytic stability of CoFe_2_O_4_–SiC

**Figure 9.**
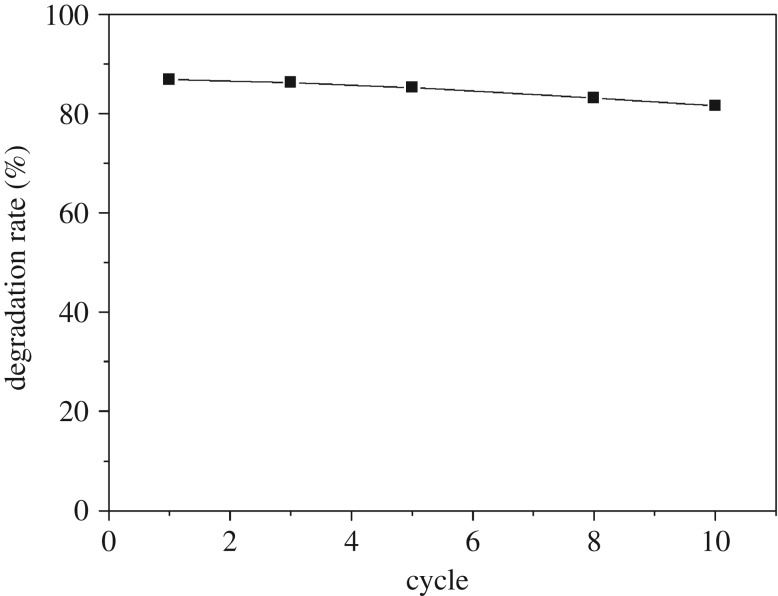
Stability of CoFe_2_O_4_–SiC foam.

The stability and recyclability of a catalyst are very important in practical applications. In this study, the used CoFe_2_O_4_–SiC foam was taken out, washed several times with ultrapure water, dried at 80°C for 4 h and then reused under MW irradiation. As shown in figure 9, after 10 consecutive runs, the degradation rate remained as high as 81.53%, indicating that the CoFe_2_O_4_–SiC possesses good stability in the MW-assisted catalytic degradation reaction. Zhang [20] synthesized CFA/CFO composites, but the composite must be regenerated by the thermal method. However, the stability of the CoFe_2_O_4_–SiC foam was very good and did not need the regenerative process. Compared with the study of Gao *et al*. [16], the stability of CoFe_2_O_4_–SiC foam was better than that of MgFe_2_O_4_–SiC.

### 3.5. Activation and degradation mechanisms

#### 3.5.1. Hot spots

Unlike conventional heating methods, MW radiation permeates and heats materials at the molecular level. Specifically, the dipole molecules under MW radiation can never rotate fast enough to follow the oscillating electric field. This phenomenon leads to the thermal effect, as a large amount of energy is consumed by this type of delay [22,23]. Some particles on the catalyst surface can absorb MW radiation more strongly than other parts, reaching a temperature of 1000°C or higher [24]. These overheated particles are referred to as ‘hot spots' [25], and they have been proved to promote the degradation of organic pollutants due to the high temperature [26].

In this case, the CoFe_2_O_4_ particles (figure 2*d*) loaded on SiC foam can become much hotter than other parts. Additionally, the SiC foam is also effective in absorbing MW radiation. Therefore, when the waste water flowed into the reactor, the high temperature caused the water to vaporize, leaving MG on the catalyst surface to be degraded by CoFe_2_O_4_ particles under MW irradiation. At the same time, because the whole reaction was exposed to air, the large organic molecules were further oxidized into smaller ones.

#### 3.5.2. Hydroxyl radical as oxidation agent

The Fenton process is a typical advanced oxidation process [27], in which the main reactions can be expressed according to the following equations:

3.1 $$\begin{array}{rl} & \text{F}{\text{e}}^{2+}+{\text{H}}_{2}{\text{O}}_{2}\to \text{F}{\text{e}}^{3+}+\cdot \text{OH}+\text{O}{\text{H}}^{-}, \end{array}$$

3.2 $$\begin{array}{rl} & \text{F}{\text{e}}^{2+}+\cdot \text{OH}\to \text{F}{\text{e}}^{3+}+\text{O}{\text{H}}^{-} \end{array}$$

3.3 $$\begin{array}{rl} \;\text{and}\; & \text{F}{\text{e}}^{3+}+{\text{H}}_{2}{\text{O}}_{2}\to \text{F}{\text{e}}^{2+}+{\text{H}}_{2}\text{O}+{\text{H}}^{+}. \end{array}$$

**Figure 10.**
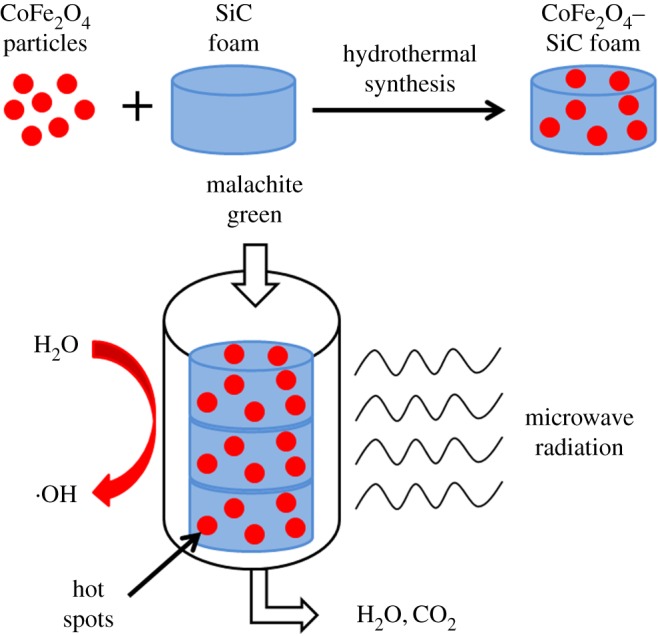
Possible mechanism of MW-induced catalytic degradation of MG.

The ·OH (figure 10) radicals produced by the Fenton process are strongly oxidizing, and therefore could be used to degrade organics. When Fe^2+^ is replaced with other species (denoted as M*^*n*^*^+1^), ·OH still can be generated from catalysing H_2_O_2_ through a slightly different Fenton-like process, as shown in the following equations [28,29]:

3.4 $${\text{M}}^{n+1}+{\text{H}}_{2}{\text{O}}_{2}\to {\text{M}}^{n+}+{\text{H}}^{+}+\cdot \text{OOH}$$

and

3.5 $${\text{M}}^{n+}+{\text{H}}_{2}{\text{O}}_{2}\to {\text{M}}^{n+1}+\text{O}{\text{H}}^{-}+\cdot \text{O}\text{H}.$$

The Fenton/Fenton-like processes are the main mechanisms for generating ·OH. When combined with MW irradiation, the process is accelerated to produce ·OH more rapidly [30]. Gao *et al*. [16] confirmed that in the MW-assisted catalytic reaction with MgFe_2_O_4_–SiC, ·OH could still be generated without adding H_2_O_2_. Therefore, in this experiment, the ·OH could be generated by MW irradiation with the CoFe_2_O_4_–SiC foam catalyst via the following equations:

3.6 $$\begin{array}{rl} & \text{F}{\text{e}}^{2+}+{\text{O}}_{2}\to \text{F}{\text{e}}^{3+}+{{\text{O}}_{2}}^{-}, \end{array}$$

3.7 $$\begin{array}{rl} & \text{F}{\text{e}}^{2+}+{{\text{O}}_{2}}^{-}+2{\text{H}}^{+}\to \text{F}{\text{e}}^{3+}+{\text{H}}_{2}{\text{O}}_{2}, \end{array}$$

3.8 $$\begin{array}{rl} & \text{F}{\text{e}}^{2+}+{\text{H}}_{2}{\text{O}}_{2}\to \text{F}{\text{e}}^{3+}+\cdot \text{OH}+\text{O}{\text{H}}^{-} \end{array}$$

3.9 $$\begin{array}{rl} \;\text{and}\; & \text{F}{\text{e}}^{2+}+\cdot \text{OH}+{\text{H}}^{+}\to \text{F}{\text{e}}^{3+}+{\text{H}}_{2}\text{O}. \end{array}$$

With the generated ·OH and H^+^, the organics in the waste water could be oxidized into small molecules [31]. Hence, there are good reasons to believe that ·OH and H^+^ are the main active species in the MW-assisted catalytic degradation of MG [32].

### 3.6. Degradation products

A variety of degradation products were identified from the collected solution after the reaction by LC-MS analysis. Electronic supplementary material, figure SF-2(a), shows the liquid chromatogram of the solution before the reaction, showing a single peak at 3.5 min. The corresponding MS peak in electronic supplementary material, figure SF-2(a), is located at *m/z* = 329, confirming that MG was the only organic matter in the original solution. In the chromatogram after the reaction (electronic supplementary material, figure SF-2(b)), in addition to the MG peak at 3.5 min, two additional peaks appeared at 6.7 and 6.8 min. The former has a formula weight of 273 according to the MS, and the intensity of the latter was very weak and could be ignored. Liu *et al*. [33] investigated the reaction intermediate in the photocatalytic oxidation of MG. Based on that study, the MG degradation process can be described by the reactions in electronic supplementary material, figure SF-3. First, MG is oxidized to dimethylnitrosamine and quinones. Indeed, a small amount of quinones was identified in electronic supplementary material, figure SF-2(b). Considering that the boiling points of the quinones are low and the temperature in the reactor was very high, some of the quinones could have volatilized into air during the experiment and were not detected by LC-MS. At the same time, most quinones were oxidized to carbon dioxide and water by oxygen.

Compared with traditional adsorption treatment of organic matter in aqueous medium, the process of MW catalytic degradation was a chemical process, and MG in aqueous medium was degraded into small molecules. Furthermore, the efficiency of MW degradation and adsorption were both very high, hence the MW catalytic degradation system could be used in practical application.

## 4. Conclusion

In this work, CoFe_2_O_4_ loaded on a SiC foam support was successfully synthesized by a hydrothermal method. The SiC skeleton effectively improved the ability to absorb MW radiation. The prepared CoFe_2_O_4_–SiC foam was shown to be a novel and efficient catalyst during the MW-assisted catalytic degradation of MG. The degradation rate reached 95.01% in less than 5 min. In addition, the effects of reaction conditions (MW power, pH, flow speed, amount of catalyst and initial MG concentration) on the degradation rate were examined. Moreover, a possible mechanism for the MW-assisted catalytic process was proposed. The temperature rise from heating by MW irradiation and the generated ·OH radicals are key for the reaction. The overall process was continuous and highly efficient, and the catalyst had good recyclability. Therefore, this new MW catalyst exhibited high degradation efficiency, broad pH range and excellent stability. It is expected to have great potential in applications of waste water treatment.

## Supplementary Material

Supplementary figures

## Data accessibility

This article does not contain any additional data.
