# Supplementary material for: Rapid degradation of malachite green by CoFe2O4–SiC foam under microwave radiation
Source: R Soc Open Sci. 2018 Jun 27;5(6):180085. doi: 10.1098/rsos.180085 (PMC6030342; doi:10.1098/rsos.180085)
Supplement: Supplementary figures [file rsos180085supp1.docx]

**Supplementary figures to the manuscript entitled**

**Rapid degradation of malachite green by CoFe_2_O_4_-SiC foam under microwave radiation**

Yanpeng Mao^†*^, Shanxiu Yang^†^, Chao Xue, Miaomiao Zhang, Wenlong Wang, Zhanlong Song, Xiqiang Zhao, Jing Sun

National Engineering Laboratory of Coal-fired Pollutants Emission Reduction, School of Energy and Power Engineering, Shandong University, Jinan 250100, P. R. China

Submitted to Royal Society Open Science

on 17^th^ January

*Corresponding author. Phone +86 531 88399372, Fax +86 531 88395877. E-mail: [maoyanpeng@sdu.edu.cn](mailto:maoyanpeng@sdu.edu.cn).

† These authors contributed equally to this work.














Figure SF-1. XPS spectra of (a) C, (b) Co, (c) Fe, (d) Si, (e) O.


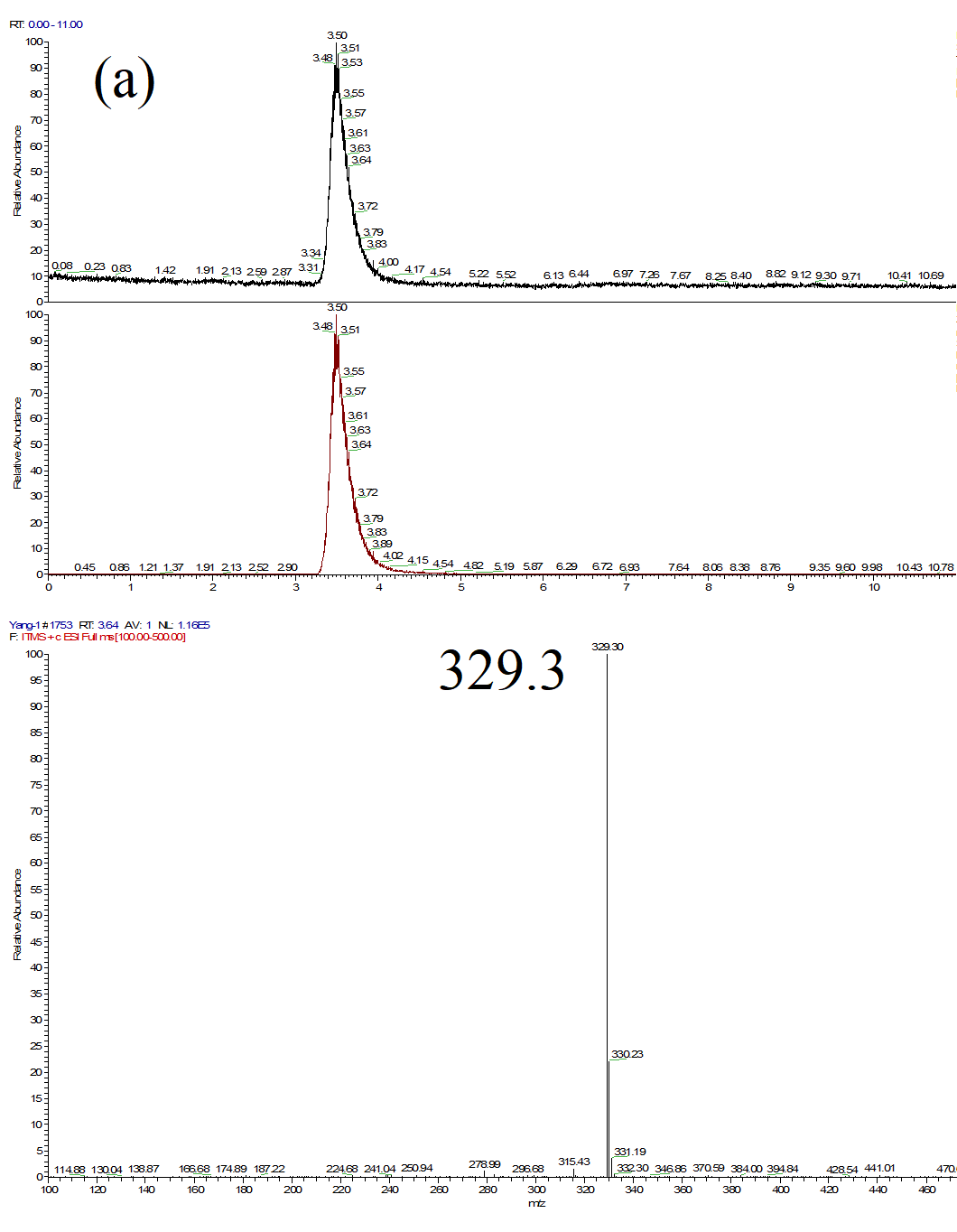

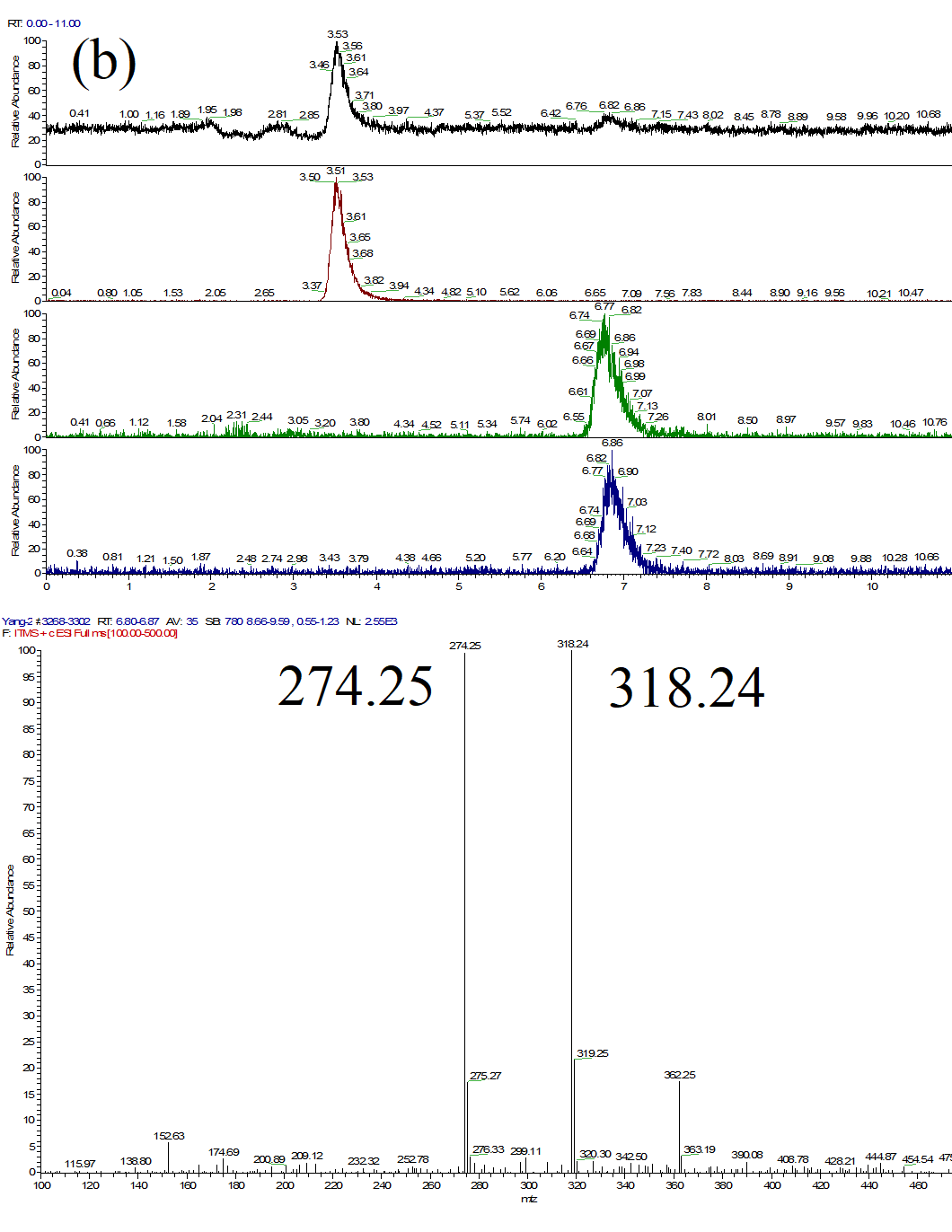


Figure SF-2. (a)LC-MS chromatograms of MG solution before the reaction; (b) LC-MS chromatograms of MG solution after reaction.


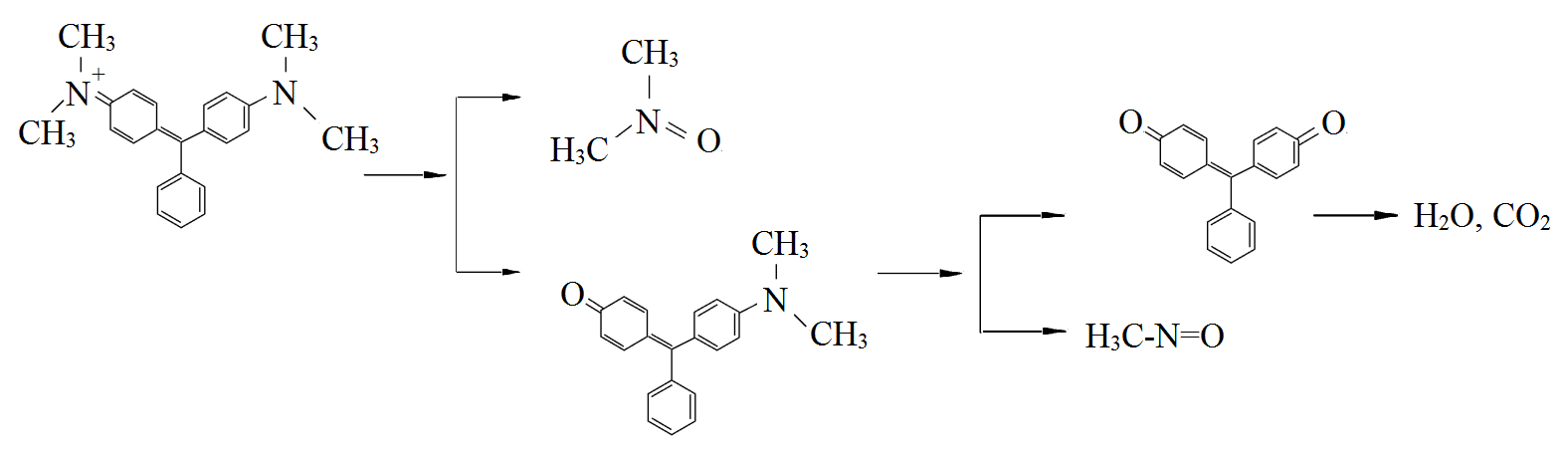


Figure SF-3. Proposed reaction scheme for the degradation of MG in the MW-catalytic process.
